# Supplementary material for: Pinpointing the tumor-specific T cells via TCR clusters
Source: eLife. 2022 Apr 4;11:e77274. doi: 10.7554/eLife.77274 (PMC9023053; doi:10.7554/eLife.77274)
Supplement: Supplementary file 1. [file elife-77274-supp1.docx]

**Supplementary File 1**. Patients and clinical characteristics.

| **patient ID** | **gender** | **age** | **BRAFmut** | **HLA-A*02** | **diagnosis** |
| --- | --- | --- | --- | --- | --- |
| mp24 | F | 53 | V600E |  | melanoma |
| mp26 | F | 67 | wt | + | melanoma |
| mp32 | F | 65 | V600E |  | melanoma |
| mp34 | F | 55 | V600E |  | melanoma |
| mp35 | F | 54 | V600E |  | melanoma |
| mp36 | F | 61 | V600E |  | melanoma |
| mp39 | F | 61 | wt |  | melanoma |
| mp41 | F | 57 | wt | + | melanoma |
| mp42 | F | 75 | N/A |  | melanoma |
| mp44 | F | 62 | wt | + | melanoma |
